# Potential therapeutic targets for pelvic organ prolapse: insights from key genes related to blood vessel development

**DOI:** 10.3389/fmed.2024.1435135

**Published:** 2024-07-25

**Authors:** Huaye Wu, Lu Yang, Jiakun Yuan, Ling Zhang, Qin Tao, Litong Yin, Xia Yu, Yonghong Lin

**Affiliations:** ^1^Department of Obstetrics and Gynecology, Chengdu Women's and Children's Central Hospital, School of Medicine, University of Electronic Science and Technology of China, Chengdu, China; ^2^Department of Clinical Laboratory, Chengdu Women's and Children's Central Hospital, School of Medicine, University of Electronic Science and Technology of China, Chengdu, China

**Keywords:** pelvic organ prolapse, blood vessel development-related genes, extracellular matrix, HAS2, MMP19, PLXDC1

## Abstract

**Objective:**

Pelvic organ prolapse (POP) is a disease in which pelvic floor support structures are dysfunctional due to disruption of the extracellular matrix (ECM). The vascular system is essential for maintaining ECM homeostasis. Therefore, this study explored the potential mechanism of blood vessel development-related genes (BVDRGs) in POP.

**Methods:**

POP-related datasets and BVDRGs were included in this study. Differentially expressed genes (DEGs) between the POP and control groups were first identified in the GSE12852 and GSE208271 datasets, and DE-BVDRGs were identified by determining the intersection of these DEGs and BVDRGs. Subsequently, the feature genes were evaluated by machine learning. Feature genes with consistent expression trends in the GSE12852 and GSE208271 datasets were considered key genes. Afterward, the overall diagnostic efficacy of key genes in POP was evaluated through receiver operating characteristic (ROC) curve analysis. Based on the key genes, enrichment analysis, immune infiltration analysis and regulatory network construction were performed to elucidate the molecular mechanisms underlying the functions of the key genes in POP.

**Results:**

A total of 888 DEGs1 and 643 DEGs2 were identified in the GSE12852 and GSE208271 datasets, and 26 candidate genes and 4 DE-BVDRGs were identified. Furthermore, Hyaluronan synthase 2 (HAS2), Matrix metalloproteinase 19 (MMP19) and Plexin Domain Containing 1 (PLXDC1) were identified as key genes in POP and had promising value for diagnosing POP (AUC > 0.8). Additional research revealed that the key genes were predominantly implicated in immune cell activation, chemotaxis, and cytokine release via the chemokine signaling pathway, the Nod-like receptor signaling pathway, and the Toll-like receptor signaling pathway. Analysis of immune cell infiltration confirmed a decrease in the proportion of plasma cells in POP, and MMP19 expression showed a significant negative correlation with plasma cell numbers. In addition, regulatory network analysis revealed that MALAT1 (a lncRNA) targeted hsa-miR-503-5p, hsa-miR-23a-3p and hsa-miR-129-5p to simultaneously regulate three key genes.

**Conclusion:**

We identified three key BVDRGs (HAS2, MMP19 and PLXDC1) related to the ECM in POP, providing markers for diagnostic studies and investigations of the molecular mechanism of POP.

## Introduction

Pelvic organ prolapse (POP) is a widespread condition that is associated with symptoms such as bulging of the anterior and posterior vaginal walls, uterine prolapse, and rectal bladder prolapse. This condition can significantly impact women’s quality of life during their reproductive and menopausal years. The lifetime risk of surgery for POP is 12.6%, and given the aging population in the United States, surgery rates for POP are expected to increase by 48% by 2050 (1). While surgery can achieve anatomical reduction, the reoperation rate is approximately 20%, and complications such as mesh exposure and recurrence are still possible (2, 3). Therefore, understanding the mechanism underlying POP is crucial for developing new therapeutic approaches.

POP is often associated with multiple pregnancies and childbirth, during which the uterus and vagina undergo significant pressure and stretching (4). This can lead to damage to the supporting structures of the pelvic floor, resulting in the relaxation of pelvic floor muscles and fascia, thereby compromising the support and fixation function of pelvic organs in their normal positions. During the pathological process of POP, the extracellular matrix (ECM), which is an indispensable component of the pelvic fascial tissue structure, forms a sophisticated network comprising proteins and polysaccharides (5). The ECM serves as the bedrock for the pelvic fascial system, enabling resistance against physiological loads and maintenance of organ stability. Moreover, the ECM actively regulates cellular behavior and function, exerting a pivotal effect on the pelvic fascial system. However, the function of the ECM transcends mere passive support; it engages with cell surface receptors, transmitting indispensable biochemical signals that are vital for preserving normal cell function and adapting to fluctuations in external pressure. When the ECM sustains damage or undergoes compositional alterations, such as heightened degradation or diminished collagen synthesis, it may compromise the supportive capacity of the pelvic fascial system. Consequently, this can result in diminished tissue elasticity and strength, thereby increasing susceptibility to POP (6, 7). Previous studies have shown that estrogen reduction, the inflammatory response and other signaling pathways may be involved (8–11). However, another issue of great interest is the possible influence of blood vessels on the pathogenesis of POP has not yet been reported. The evolution of complex multicellular organisms depends on the development of transport systems to fulfill the metabolic needs of cells across various organs, body types, and sizes. In vertebrates, a closed vascular system is essential for this purpose (12). The network of blood vessels plays a crucial role in the body by carrying oxygen, nutrients, and waste products as well as facilitating communication between organs and tissues. Studies have indicated that utilizing tissue engineering techniques to implant tissue-engineered mesh in rat models can improve angiogenesis (13), and transplanting mesenchymal stem cells can enhance healing in aged rats with vaginal wall incisions by promoting angiogenesis, reducing inflammation and decreasing the expression of matrix metalloproteinase 19 (MMP19) (14). Therefore, exploring the blood vessel development-related genes (BVD-RGs) that are associated with POP may be another way to identify therapeutic targets for POP.

To address this issue, in this research, we investigated the potential role of BVD-RGs as essential genes in POP and their underlying molecular mechanisms. This was achieved by retrieving BVD-RGs and conducting various bioinformatics analyses on POP, with the goal of providing a new perspective for the clinical management of POP.

## Materials and methods

### Preparation of relevant data

The GSE12852, GSE28660, GSE208271 and GSE220515 POP-related datasets were extracted from the Gene Expression Omnibus (GEO) database^1^ (Table 1). In addition, the Gene Ontology (GO) database^2^ was searched for the GO:0001568 pathway with the keyword “blood vessel development,” and 520 BVDRGs were extracted for this research (Supplementary material).

**Table 1:** POP-related datasets.

Datasets	Source	Data type	Tissue type	Sample number	Year	Reference
GSE12852	GEO	Microarray	Round ligament, uterosacral ligament (human)	8/9 (POP vs. Control)	2008	PMID: 19056808
GSE28660	GEO	Microarray	Uterosacral ligament (human)	8/4 (POP vs. Control)	2019	PMID: 37266648
GSE208271	GEO	High throughput sequencing	Anterior vaginal wall (human)	6/6 (POP vs. Control)	2022	PMID: 36176289
GSE220515	GEO	High throughput sequencing	Vaginal wall (rats)	8/8 (POP vs. Control)	2023	PMID: 37424873

### Identification of differentially expressed genes (DEGs) in the GSE12852 and GSE208271 datasets

Differentially expressed genes (DEGs1 and DEGs2) between POP and Control samples in the GSE12852 and GSE208271 datasets were analyzed by Limma (version 3.48.3) (15) and DESeq2 (version 1.36.0) (16), respectively. The screening criteria were |log2FoldChange (FC)| > 0.5 and *p* < 0.05. The DEGs1 and DEGs2 were identified by the ggplot2 package (version 3.3.5) (17) and pheatmap package (version 1.0.12) (17). Candidate genes were derived from the intersection of downregulated and upregulated DEGs in the two datasets.

### Functional analysis of candidate genes

For the purpose of examining the physiological functions and pathways of signaling pathways associated with the candidate genes, enrichment analyses of the candidate genes for GO and Kyoto Encyclopedia of Genes and Genomes (KEGG) were performed utilizing clusterProfiler (version 4.0.2) (18) with a threshold of *p* < 0.05 and a count ≥1. The results of the enrichment analysis were analyzed by ggplot2 (version 3.3.5) (17).

### Identification of differentially expressed BVDRGs (DE-BVDRGs)

The overlapping genes between the candidate genes and the BVD-RGs were labeled DE-BVDRGs, and a gene–gene interaction (GGI) network was established through the GeneMANIA database^3^ to reveal functional genes and pathways associated with the DE-BVDRGs. Furthermore, the DE-BVDRGs in the GSE12852 dataset were subjected to Spearman correlation analysis using the ggplot2 package.

### Identification of key genes and assessment of diagnostic efficacy

Initially, to screen feature genes, two algorithms were employed, namely, the least absolute shrinkage and selection operator (LASSO) algorithm in glmnet (version 4.1–4) (19) and the support vector machine-recursive feature elimination (SVM-RFE) algorithm (20). In the LASSO and SVM-RFE algorithms, genes that overlapped were designated as candidate key genes. Subsequently, verification of the expression trends of the candidate key genes in the GSE12852 and GSE208271 datasets was performed. In this study, key genes were defined as those that exhibited consistent expression trends and demonstrated significant expression within two datasets. The diagnostic efficacy of the key genes was assessed using receiver operating characteristic (ROC) analysis in the GSE12852 and GSE208271 datasets with pROC (version 1.18.0) (21).

### Exploration of signaling pathways of key genes

Initially, the median expression values of the key genes were utilized to classify all samples in the GSE12852 dataset into high- and low-expression groups. Subsequently, the clusterProfiler (version 4.0.2) (18) and the org.Hs.eg.db (version 3.13.0) (22) packages were utilized to conduct single-gene gene set enrichment analysis (GSEA) with |NES| > 1, NOM *p* < 0.05 and *q* < 0.25. The background gene sets were KEGG (c2.cp.kegg.v7.4.entrez.gmt) and GO (c5.go.v7.4.entrez.gmt) from the Molecular Signatures Database (MSigDB). Each key gene was enriched in the top 10 pathways to determine the intersection, and the Spearman correlation was used to analyze the correlation between key genes and intersecting pathways.

### Analysis of the immune microenvironment in POP

The CIBERSORT algorithm (23) was applied to each sample of the GSE12852 dataset to determine the proportion of immune cells present. The Wilcoxon rank-sum test was used to analyze the differences in immune cell infiltration between the POP and Control groups. Next, a heatmap illustrating the correlation between key genes and immune cells was generated using Spearman correlation to analyze the relationship between the two variables.

### Regulatory network construction and drug prediction

To construct a comprehensive competing endogenous RNAs (ceRNA) regulatory network revealing the regulatory relationships of key genes, the miRWalk, miRDB and miRTarBase databases were used. The miRWalk and miRDB provided predictions of miRNAs that regulate key genes, while the miRTarBase database offered predictions of lncRNAs that target miRNAs. Cytoscape software (version 3.8.2) was used for visualization of ceRNA regulatory networks. Transcription factors (TFs) of key genes were identified, and TF-mRNA networks were visualized with the help of the Cistrome database (RP-score > 0.8).

Additionally, the expression of key genes at the tissue-organ level was investigated by querying the BioGPS database^4^ for key gene distribution data in organs and tissues. Then, the average value of key genes in each tissue was calculated, the relevant organs and tissues whose expression of key genes was greater than the overall average were selected, and the tissue-key gene network map was drawn with Cytoscape software.

The GeneCrad database^5^ was used to predict possible drugs that could be used for the treatment of POP and to construct key gene-drug interaction networks.

### Expression patterns of key genes

The expression of important genes was considered in the GSE28660, GSE208271, and GSE220515 datasets by applying the Wilcoxon rank-sum test to determine the expression of key genes in the uterosacral ligament tissue of POP patients and control uterosacral ligament tissue samples.

### Statistical analysis

The statistical analysis was conducted using the R programming language (version 4.1.0). To analyze the data across different categories, the Wilcoxon test was utilized. Unless otherwise specified, statistical significance was defined as a *p* value less than 0.05.

## Results

### A total of 26 candidate genes were obtained

**Figure 1:**
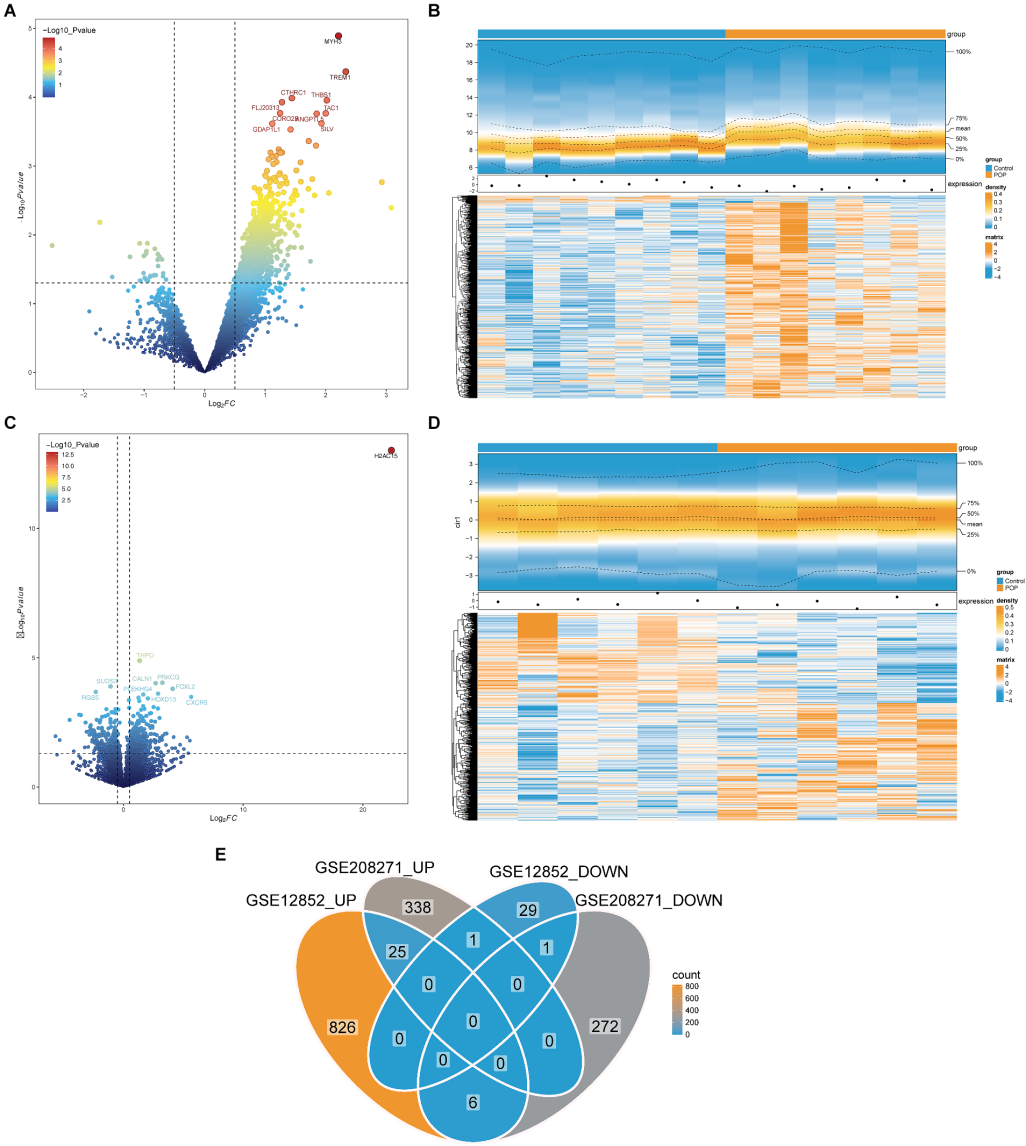
Identification of candidate genes in the GSE12852 and GSE208271 datasets. **(A,B)** Volcano and heat maps of Differentially expressed genes 1 (DEGs1) between POP and control groups in the GSE12852 dataset. **(C,D)** Volcano and heat maps of Differentially expressed genes 2 (DEGs2) between POP and control groups in the GSE208271 dataset. **(E)** Totally 26 candidate genes were obtained by taking the intersection of DEGs1 and DEGs2.

A total of 888 DEGs1 were identified in the GSE12852 dataset, comprising 857 upregulated DEGs1 and 31 downregulated DEGs1 (Figures 1A,B). Similarly, 643 DEGs2 were identified in GSE208271, including 364 upregulated DEGs2 and 279 downregulated DEGs2 (Figures 1C,D). Subsequently, out of the two datasets, a total of 26 candidate genes were identified by locating the shared genes that were both upregulated and downregulated. Among the majority of these genes, 25 exhibited a substantial increase in expression in samples from patients with POP, and 1 gene exhibited a significant decrease in expression in POP patients (Figure 1E).

### Candidate genes were mainly involved in the regulation of the ECM, protein kinases and cytokines

**Figure 2:**
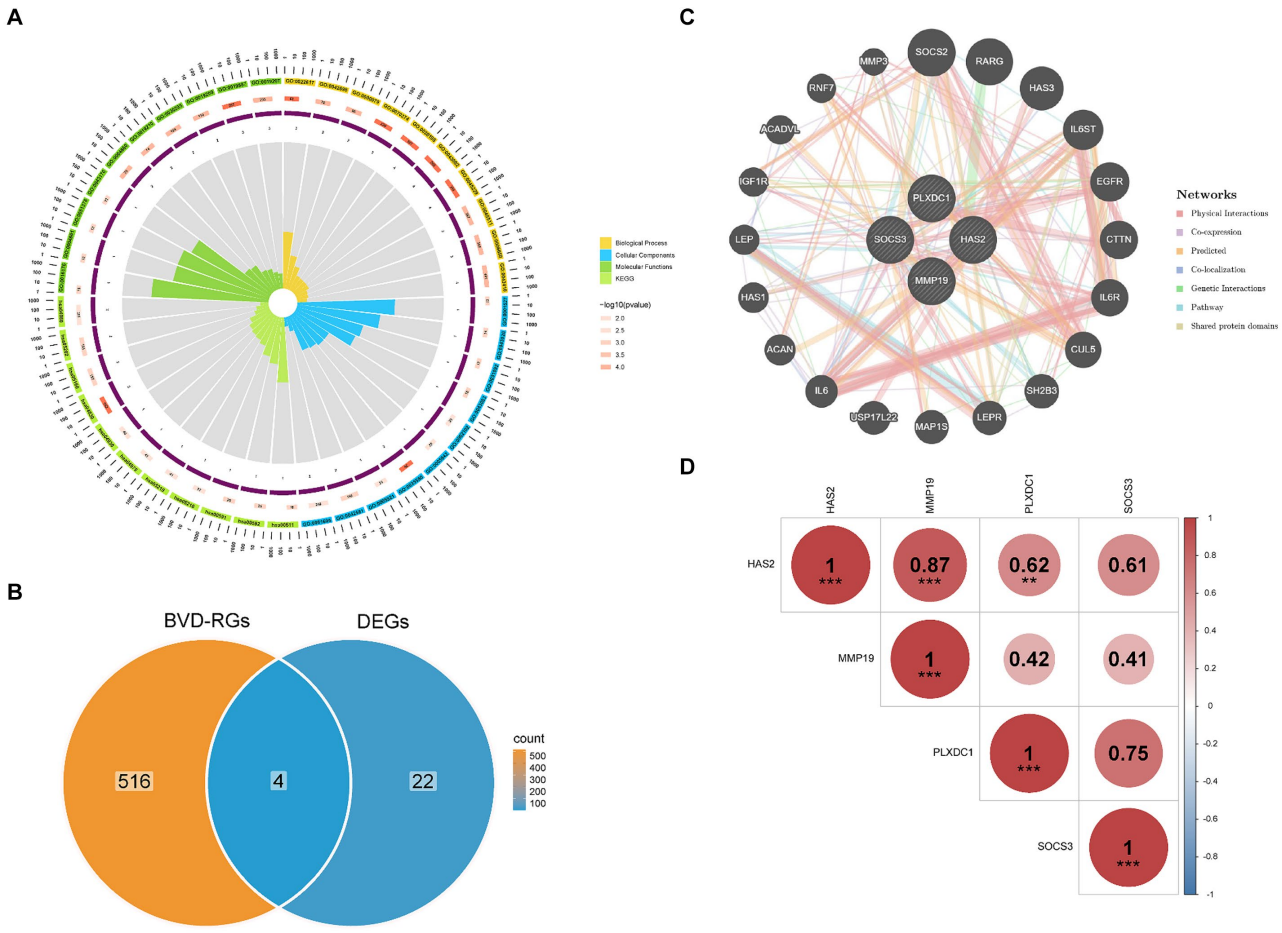
Functional enrichment of candidate genes and identification of differentially expressed BVDRGs (DE-BVDRGs). **(A)** Analysis of biological processes and signalling pathways involved in candidate genes. **(B)** Identification of DE-BVDRGs. **(C)** Construction of a gene–gene interaction (GGI) network revealed interactions between DE-BVDRGs. **(D)** Correlation analysis revealed correlation between DE-BVDRGs.

The 26 candidate genes were enriched in numerous GO items such as ECM disassembly, specific granule lumen and protein kinase regulator activity. In addition, the signaling pathways primarily associated with the candidate genes were the JAK–STAT signaling pathway and cytokine–cytokine receptor interaction. Consequently, the biological processes involving the candidate genes were principally associated with the regulation of the ECM and protein kinases, whereas the signaling pathways were mainly associated with the regulation of cytokines (Figure 2A).

### HAS2, MMP19, PLXDC1, and SOCS3 were identified as DE-BVDRGs with significant correlations

Candidate genes and BVD-RGs were overlapped to identify four DE-BVDRGs: MMP19, Hyaluronan synthase 2 (HAS2), Plexin Domain Containing 1 (PLXDC1), and Suppressor of Cytokine Signaling 3 (SOCS3) (Figure 2B). Then the GGI network revealed strong interactions between HAS2, MMP19, PLXDC1, SOCS3, SOCS2, RARG, HAS3, IL6ST and CTTN (Figures 2B,C). Moreover, MMP19 presented a significant positive correlation with HAS2 (|r| = 0.87, *p* < 0.05) and PLXDC1 (|r| = 0.62, *p* < 0.05) (Figure 2D).

### HAS2, MMP19 and PLXDC1 were identified as key genes with favorable diagnostic efficacy

**Figure 3:**
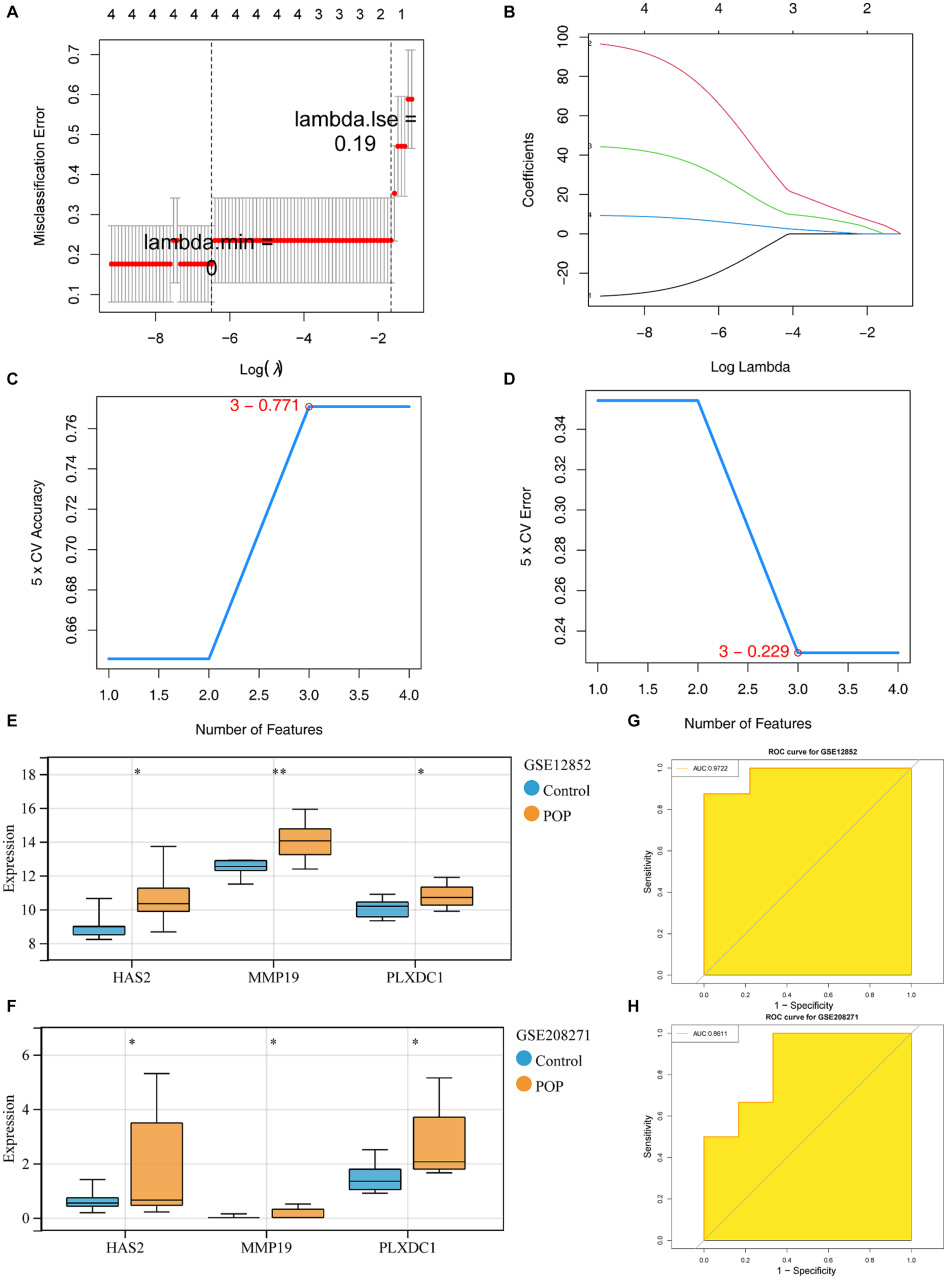
Identification and diagnostic value assessment of three key genes. **(A,B)** Regression coefficient path diagram and cross-validation curves in least absolute shrinkage and selection operator (LASSO) logistic regression algorithm. **(C,D)** The curve of change in the predicted true and error value of each gene in support vector machine - recursive feature elimination (SVM-RFE) algorithm. **(E,F)** Expression difference of the three key genes in POP and control groups within GSE12852 and GSE208271 datasets,respectively. **(G,H)** The receiver operating characteristic (ROC) curve of the three key genes in GSE12852 and GSE208271 datasets.

First, 4 feature genes, namely, HAS2, MMP19, PLXDC1 and SOCS3 (lambda.min = 0), were selected by LASSO analysis (Figures 3A,B). Next, the SVM-RFE model was constructed to screen the feature genes. At the highest accuracy (0.771) and lowest error rate (0.229), 3 feature genes (MMP19, PLXDC1 and HAS2) were collectively identified (Figures 3B–D). Finally, the feature genes screened by LASSO and SVM-RFE were overlapped to identify 3 candidate key genes (MMP19, PLXDC1 and HAS2). The expression patterns of the candidate key genes were confirmed in the GSE12852 and GSE208271 datasets, revealing enormous increases in the expression of HAS2, MMP19, and PLXDC1 in the POP population (*p* < 0.05). Therefore, these genes were defined as key genes in this study (Figures 3E,F). Moreover, the key genes had the ability to discriminate POP patients from control individuals in the GSE12852 and GSE208271 datasets (AUC > 0.8) (Figures 3G,H).

### Key genes promoted POP progression through immune cell activation and chemotaxis

**Figure 4:**
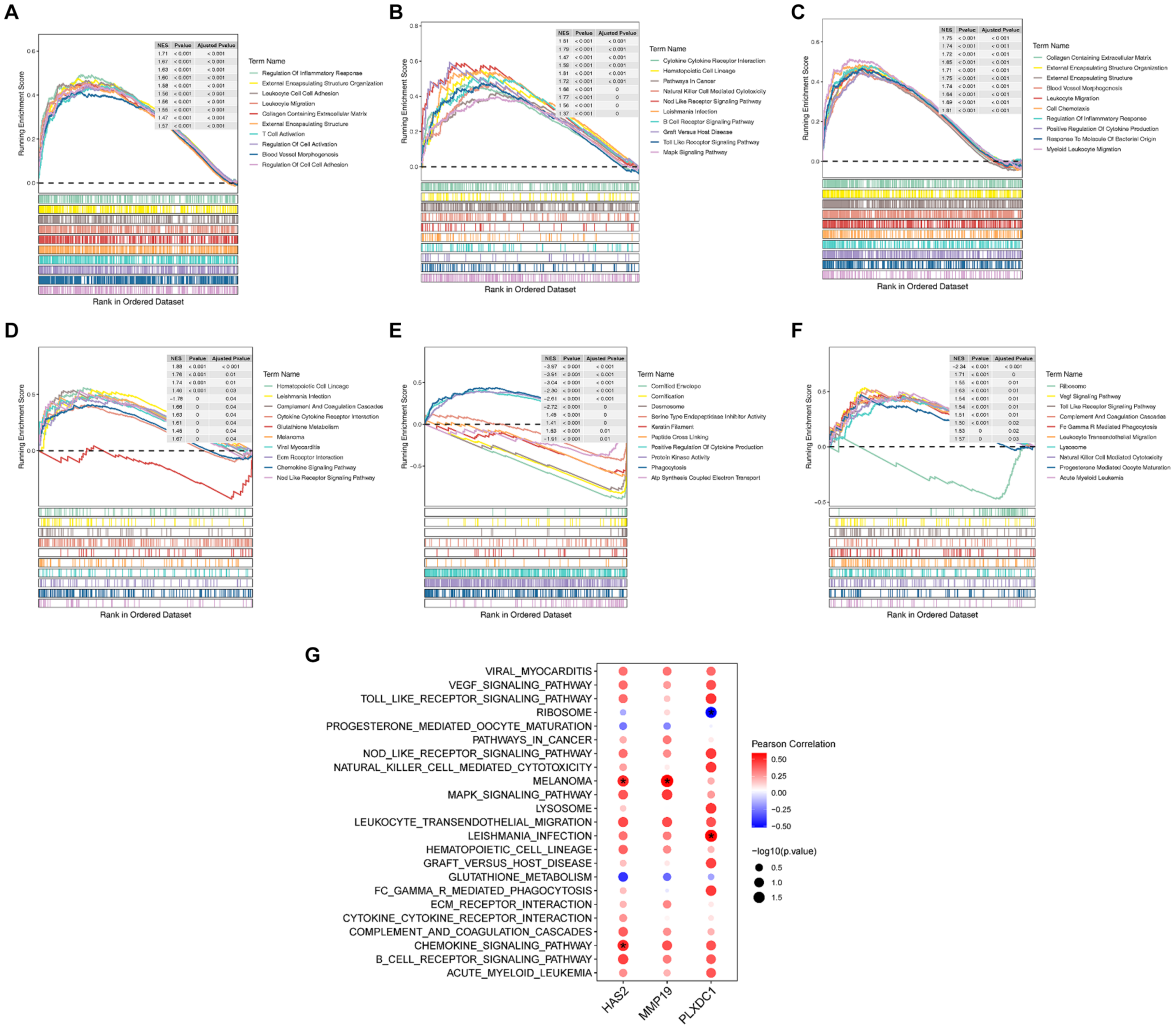
Enrichment analysis of key genes. **(A–F)** Results of Gene Set Enrichment Analysis (GSEA) for key genes, showing TOP10 GO entries and TOP10 KEGG items. **(G)** Correlation analysis of key genes and functional items.

HAS2 and MMP19 were implicated in functional processes associated with immune cell activation, chemotaxis, and release of cytokines, according to the GSEA results. The chemokine signaling pathway, leukocyte migration pathway, cell chemotaxis pathway, inflammatory response regulation pathway, positive regulation of cytokine production pathway, cytokine–cytokine–receptor interaction pathway, and Nod-like receptor signaling pathway are all examples of such processes (Figures 4A–D). Similarly, PLXDC1 is involved in the positive regulation of cytokine production, phagocytosis, the VEGF signaling pathway, the Toll-like receptor signaling pathway, FC-γR-mediated phagocytosis, leukocyte transendothelial migration, natural killer cell-mediated cytotoxicity and progesterone-mediated oocyte maturation (Figures 4E,F). Additionally, a correlation analysis revealed significant positive correlations between HAS2 and chemokine signaling pathways (*r* = 0.505, *p* = 0.039) and melanoma (*r* = 0.510, *p* = 0.037). MMP19 was strongly associated with melanoma (*r* = 0.593, *p* = 0.012), while PLXDC1 was associated with Leishmania infection (*r* = 0.581, *p* = 0.014) and ribosomes (*r* = −0.517, *p* = 0.033) (Figure 4G).

### Plasma cells and key genes coordinated in the progression of POP

**Figure 5:**
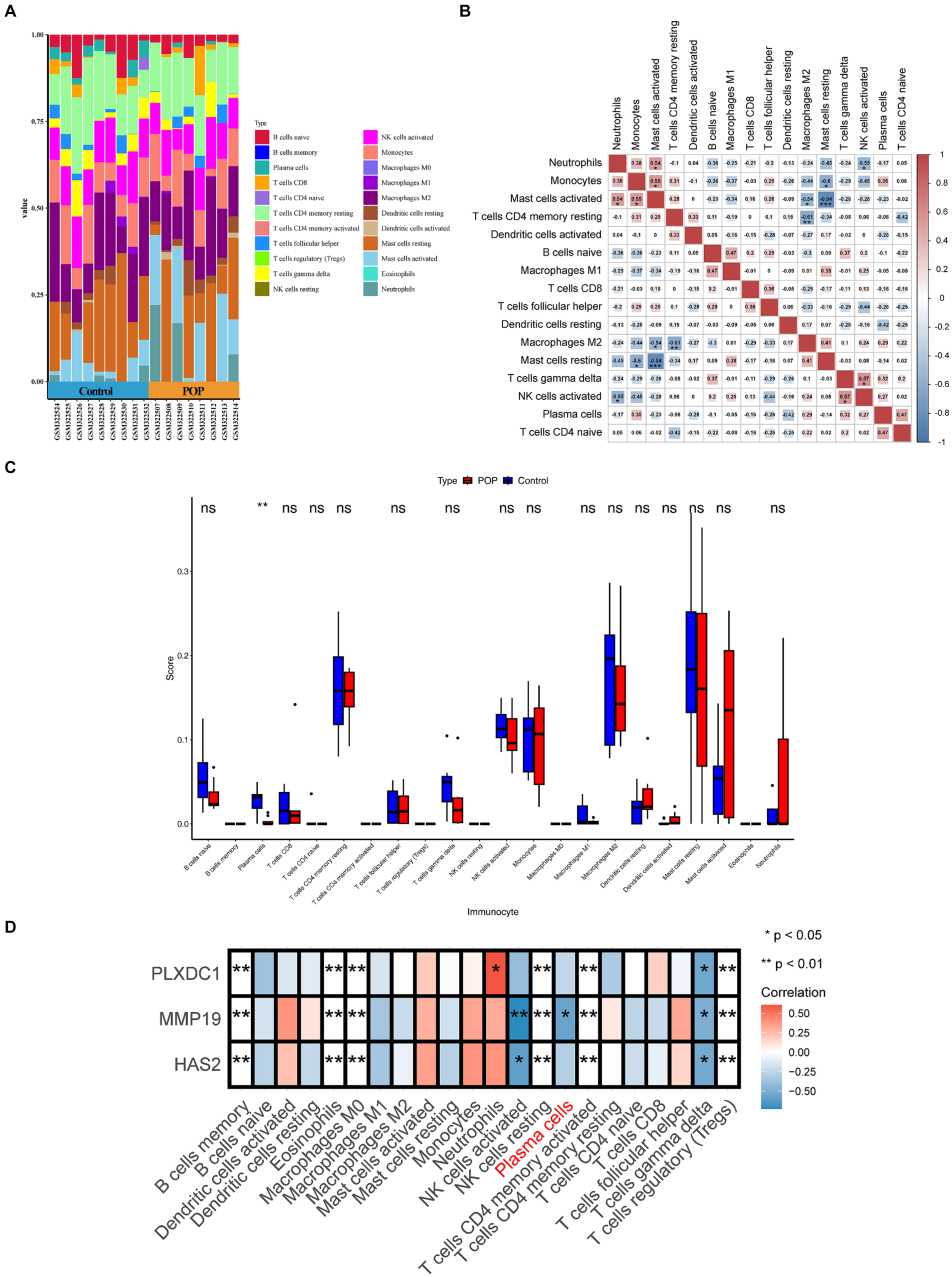
Evaluation and visualization of immune cell infiltration. **(A)** Stacked histogram comparing control and POP samples for the immune cell percentage. **(B)** Correlation heat map of immune cells. **(C)** Histogram showing 22 types of immune cells in proportion between POP and control. Data were analyzed by Wilcoxon test. **(D)** Heatmap displaying correlation between key genes and immune cells.

Immune cell activation, chemotaxis, and cytokine secretion were associated with key genes; therefore, an immunocellular infiltration analysis was conducted on POP samples. However, the results revealed decreased infiltration of plasma cells in POP patients (*p* < 0.05), and the correlation between MMP19 expression and plasma cell numbers was significantly negative (*r* = −0.569, *p* = 0.017). Furthermore, there was a significant association between the expression of PLXDC1 and the number of neutrophils (*r* = 0.605, *p* < 0.05) (Figures 5A–D).

### Analysis of key gene regulatory networks

**Figure 6:**
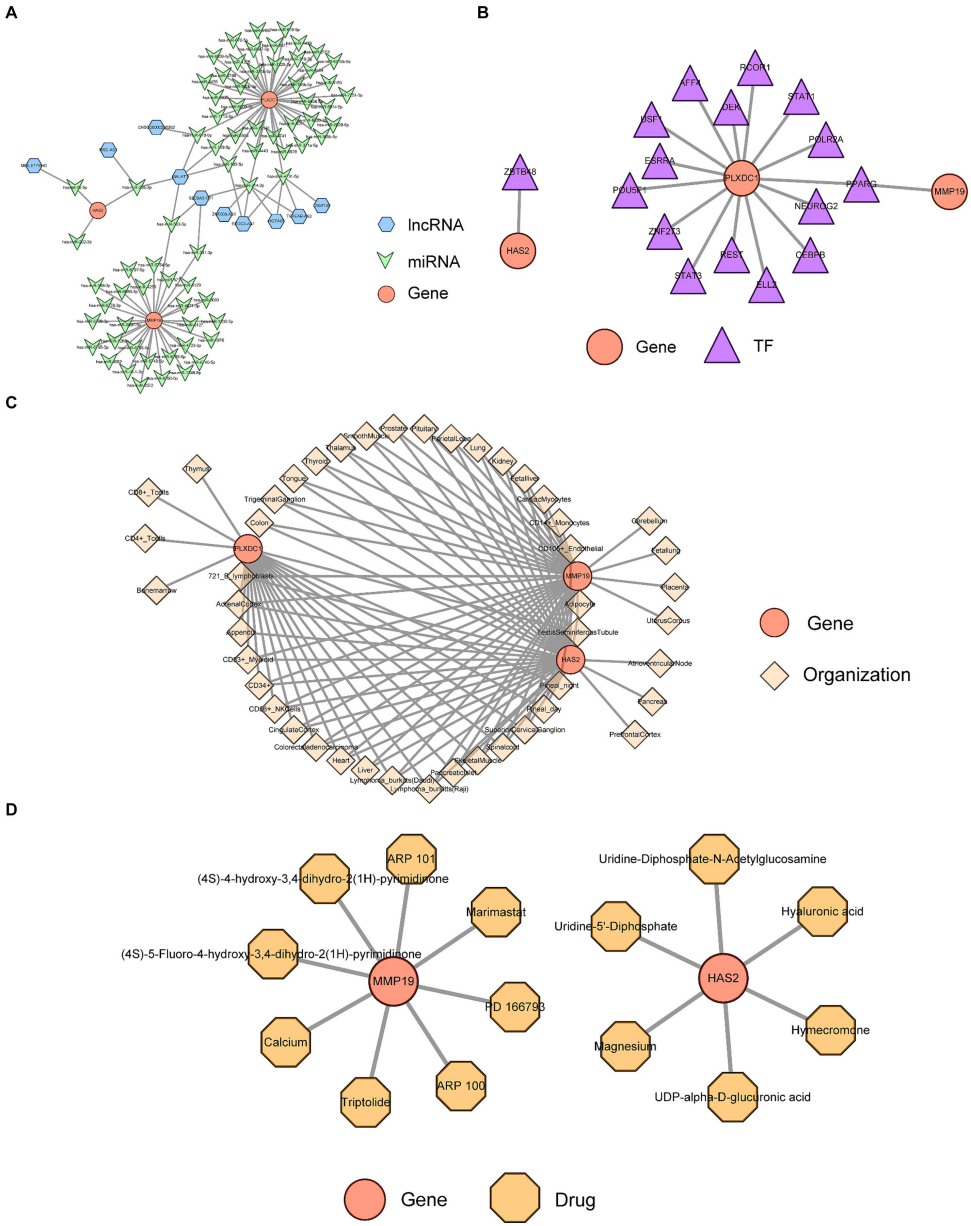
Analysis of key gene regulatory networks. **(A)** The Competing endogenous RNA (ceRNA) regulatory network of key genes. **(B)** Transcription factors (TFs) prediction for key genes. **(C)** Expression and distribution of key genes in organs and tissues. **(D)** Prediction of targeted drugs for key genes.

The ceRNA regulatory network revealed that MALAT1 (a lncRNA) targets hsa-miR-503-5p, hsa-miR-23a-3p and hsa-miR-129-5p to simultaneously regulate three key genes (Figure 6A). A TF-key gene regulatory network revealed that PPARG regulated the transcription of PLXDC1 and MMP19, while ZBTB48 regulated HAS2 (Figure 6B). The key gene-organ network indicated that key genes were differentially expressed in different organs. Specifically, HAS2 is expressed in the heart, liver, Burkitt’s lymphoma cells (Raji cells), and skeletal muscle. MMP19 was highly expressed in adipocytes, heart, liver, and Burkitt’s lymphoma cells (Raji cells), and PLXDC1 was highly expressed in CD4+ T cells, CD56+ NK cells, CD8+ T cells, thymus cells and pineal cells (Figures 6B,C). The key gene-drug network evidence for hyaluronic acid, magnesium and hymecromone targeted HAS2 for the treatment of POP, and marimastat, triptolide and calcium targeted MMP19. However, PLXDC1 failed to predict the target drug (Figure 6D, Table 2).

**Table 2:** Results of drug predictions.

Gene	Drug name	Sources
HAS2	Hyaluronic acid	Approved, Vet_approved
HAS2	Magnesium	Approved, Experimental, Investigational
HAS2	Hymecromone	Experimental, Investigational
HAS2	UDP-alpha-D-glucuronic acid	Experimental
HAS2	Uridine-5’-Diphosphate	Experimental
HAS2	Uridine-Diphosphate-N-Acetylglucosamine	Experimental
MMP19	(4S)-4-hydroxy-3,4-dihydro-2(1H)-pyrimidinone	Experimental
MMP19	(4S)-5-Fluoro-4-hydroxy-3,4-dihydro-2(1H)-pyrimidinone	Experimental
MMP19	Marimastat	Investigational
MMP19	Triptolide	Investigational
MMP19	Calcium	/
MMP19	ARP 100	/
MMP19	ARP 101	/
MMP19	PD 166793	/

### PLXDC1 expression was significantly upregulated in POP patients in the GSE28660 and GSE220515 datasets

**Figure 7:**
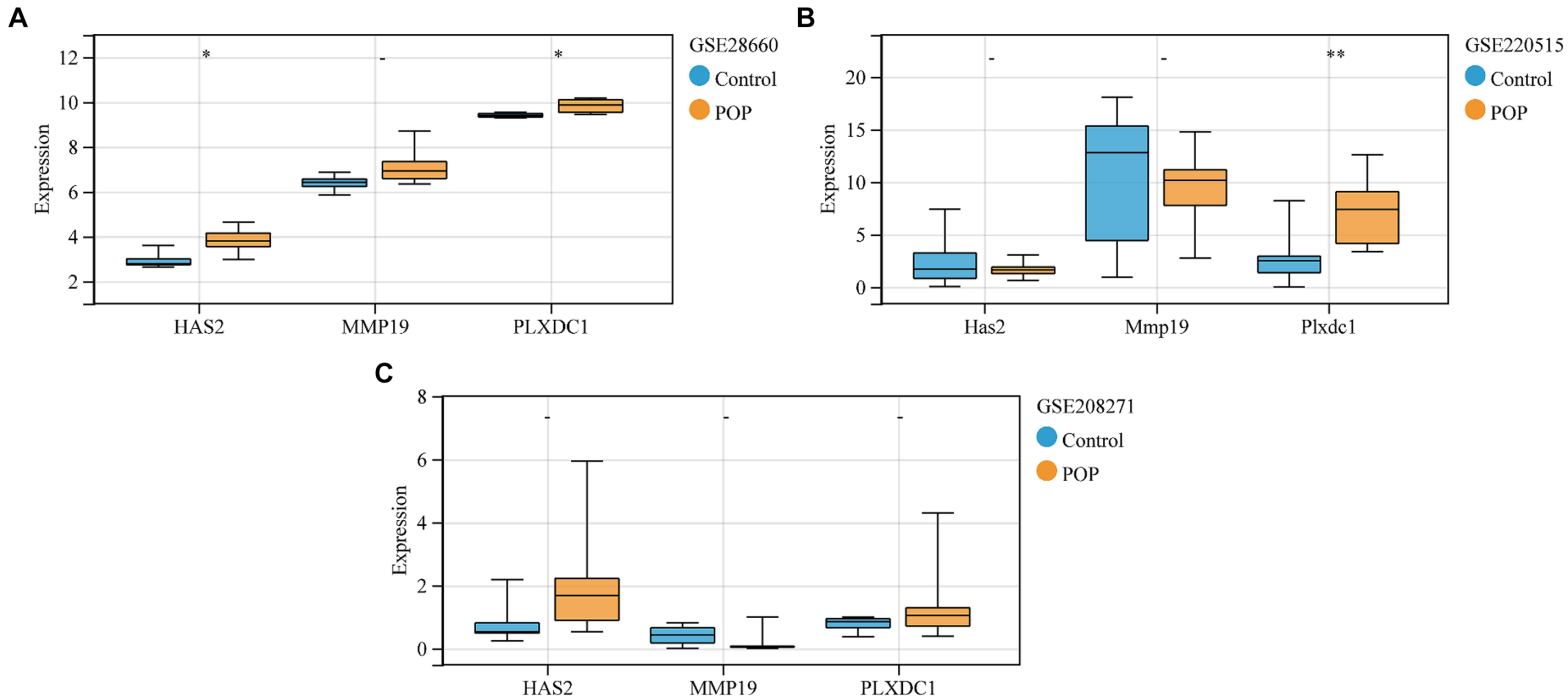
Validation of expression levels of key genes between control and POP groups. **(A–C)** Validation of expression levels of key genes in GSE28660, GSE208271 and GSE220515, respectively. Data were compared using the Wilcoxon test.

Validation of the expression trends of key genes was performed on the GSE28660, GSE208271, and GSE220515 datasets. The expression of the HAS2 and PLXDC1 genes was significantly higher in POP patients in the GSE28660 dataset, and PLXDC1 expression was significantly increased in POP patients in the GSE220515 dataset. However, there was no statistically significant difference in the expression of key genes between the POP and control groups in the GSE208271 dataset (Figures 7A–C).

## Discussion

In this study, we conducted a differential expression analysis on two datasets and identified 26 overlapping differentially expressed genes related to transcriptional dysregulation, cytokine–cytokine receptor interactions, and fat digestion and absorption. We then focused on vascular development-related genes and identified 4 differentially expressed genes (MMP19, HAS2, PLXDC1, and SOCS3). Using machine learning algorithms, we identified MMP19, HAS2, and PLXDC1 as key genes with strong potential for diagnosing POP. These genes were found to be significantly elevated in POP patients. Single-gene GSEA further elucidated the functions of these key genes. Immune cell infiltration analysis revealed a significant difference in plasma cell numbers between POP and control samples, with a negative correlation between plasma cell numbers and MMP19 expression, suggesting a role for immune cells in POP. We also explored the regulatory network of key genes by analyzing differential noncoding RNAs and database predictions. Additionally, we identified potential small molecule drugs for key genes using the GeneCrad database. External data confirmed the expression of key genes in the uterosacral ligament and anterior vaginal wall tissues of POP patients. This study highlights the importance of MMP19, HAS2, and PLXDC1 as key genes in POP and provides insights into potential molecular mechanisms and treatment options for POP patients.

During the development of POP, ECM plays a crucial role in providing structural support to the pelvic fascial tissue. Additionally, the ECM actively influences cellular behavior and function, impacting the overall health of the pelvic fascial system. When the ECM is damaged or undergoes changes, such as increased degradation or decreased collagen production, it can weaken the supportive capabilities of the pelvic fascial system. This can lead to reduced tissue strength and elasticity, increasing the risk of developing POP.

MMP19 belongs to the matrix metalloproteinase (MMP) family of enzymes responsible for the degradation and remodeling of the ECM (24). MMPs play crucial roles in maintaining tissue structure integrity and promoting cell migration, tissue repair, development, and inflammatory responses. Compared to other members of the MMP family, MMP19 exhibits unique substrate specificity and regulatory mechanisms. MMP19 is expressed in various tissues, with higher levels observed in the skin, lungs, articular cartilage, and nervous system (25, 26). In POP, MMP19 is highly expressed specifically in pelvic fascial tissue, suggesting that it may play an important regulatory role in the progression of POP. MMP19 is a member of the matrix metalloproteinase family and is located mainly in the extracellular matrix. The biological function of MMP19 is mainly to degrade various components of the extracellular matrix, including type IV collagen, laminin, and nidogen, as well as the large tenascin-C isoform, fibronectin, and type I gelatin *in vitro* (27). MMP19 affects the migration of inflammatory cells and the release of inflammatory mediators. MMP19^−/−^ mice exhibit characteristics such as blocked macrophage chemotaxis, increased inflammatory susceptibility, and inability to resolve inflammatory responses, indicating that MMP19 is an important regulator of the innate immune response (28). MMP19 is also involved in the formation and regulation of angiogenesis. MMP19 specifically cleaves human plasminogen to produce three angiostatin-like fragments with molecular weights of 35, 38, and 42 kDa, which block the proangiogenic signal induced by hepatocyte growth factor (HGF) in endothelial cells (29). Therefore, in POP pelvic floor tissue, MMP19 may also participate in regulating the maintenance and repair of the normal function of the pelvic floor fascia system by acting on the ECM, thereby playing an important role in the loss of mechanical properties of the POP pelvic floor fascia system and making it a potential therapeutic target for POP treatment.

Hyaluronic acid (HA) is a unique non-sulfated glycosaminoglycan that is widely distributed in the ECM of various tissues, including the pelvic floor fascial system, where it plays a crucial role in ECM metabolism (30, 31). HA possesses a strong water-binding capacity and is capable of absorbing and retaining several times or even 1,000 of times its weight in water, providing essential hydration and lubrication to tissues (32). This characteristic is particularly important for maintaining the flexibility and elasticity of tissues such as the pelvic floor fascial system, helping protect them from physical damage, and injectable fiber hydrogel composites have been applied in POP treatment (33). HA also participates in regulating cell behavior by binding to cell surface receptors such as CD44, influencing cell proliferation, migration, and differentiation (34). This is crucial for the repair and regeneration of pelvic floor fascial tissues, especially in damaged or diseased states of the pelvic floor fascial system. HA also plays a role in regulating inflammatory and immune responses (35, 36). Low-molecular-weight HA can promote inflammation, while high-molecular-weight HA has anti-inflammatory effects, influencing the inflammatory process and tissue repair by regulating the activity of immune cells (37). Hyaluronan synthase 2 (HAS2) plays a crucial role in the synthesis of HA, thus having a profound impact on the overall structure and function of the ECM (38). HAS2 not only directly affects the production of HA but also indirectly influences the overall physical properties of the ECM, such as lubrication, hydration status, and viscoelastic properties, thereby affecting tissue biomechanical performance and cell behavior (39). By modulating the expression and activity of HAS2, tissue repair, inflammatory responses, and the progression of various diseases can be influenced, making HAS2 a potential target for various diseases, including pelvic floor disorders, arthritis, and certain cancers.

PLXDC1, also known as tumor endothelial marker 7 (TEM7), is a membrane protein expressed in various tissues and cell types. Although it was initially identified as a marker for tumor endothelial cells (40), subsequent research has shown that PLXDC1 plays important roles in both normal physiological and pathological conditions, particularly in the dynamic adjustment of the ECM and the regulation of cell behavior (41). PLXDC1 interacts with other cell surface receptors or ECM components to regulate various cellular functions, including cell migration, proliferation, differentiation, and angiogenesis. By influencing the organization and function of ECM components, PLXDC1 indirectly participates in the dynamic adjustment of the ECM. Although PLXDC1 itself does not directly participate in ECM synthesis or degradation, it can regulate the composition and physical properties of the ECM by modulating the interaction between cells and the ECM, as well as by affecting the activity of molecules directly involved in ECM remodeling, such as MMPs (42). PLXDC1 also interacts with specific signaling molecules to influence cell behavior. These signaling molecules may include growth factors, cytokines, and other types of membrane receptors, which are collectively involved in regulating cell survival, proliferation, and migration (43). Due to its ability to modulate the interaction between cells and the ECM, PLXDC1 plays a role in weakening the pelvic floor fascial system in POP, thereby reducing the mechanical properties of the pelvic floor fascial system. Because of its role in regulating ECM dynamics, angiogenesis, and cell behavior in the pelvic floor fascial system, PLXDC1 has emerged as a potential therapeutic target for POP.

SOCS3 is a member of the SOCS protein family and is responsible for regulating signaling mediated by the JAK (Janus kinase)/STAT (Signal Transducer and Activator of Transcription) pathway (44). Through this mechanism, SOCS3 participates in controlling various cellular functions, including cell growth, differentiation, migration, and inflammatory responses (45, 46). Although the primary role of SOCS3 is to regulate cellular responses by inhibiting specific signaling pathways within cells and it is not directly involved in ECM regulation, SOCS3 can modulate the activation of immune cells and the production of inflammatory factors by inhibiting the JAK/STAT signaling pathway. This regulation is crucial for limiting inflammatory responses, preventing excessive tissue damage, and preventing ECM degradation caused by inflammation. Due to its role in regulating inflammation and immune responses and its potential impact on ECM remodeling, SOCS3 also holds value as a potential therapeutic target for POP (47, 48).

This is the first study to conduct a bioinformatics analysis on the associations between genes that are involved in angiogenesis and POP target genes. However, these bioinformatics results still need to be validated through further experiments such as gene manipulation. Moreover, different levels of sequencing methods and tissue sites have a significant impact on the study’s outcomes. In summary, exploring potential pathogenic mechanisms and therapeutic targets for POP through joint analysis of public databases is feasible. In this study, MMP19, HAS2, PLXDC1, and SOCS3, which were identified for the first time in previous research on POP, may play significant roles in the loss of mechanical properties in POP. These genes have the potential to become therapeutic targets, opening new avenues for treatment.

## Data availability statement

The original contributions presented in the study are included in the article/Supplementary material, further inquiries can be directed to the corresponding authors.

## Ethics statement

This study was based on publicly available datasets. Ethical review and approval was not required for the study, in accordance with the local legislation and institutional requirements.

## Author contributions

HW: Methodology, Writing – original draft. LuY: Visualization, Writing – review & editing. JY: Visualization, Writing – review & editing. LZ: Project administration, Supervision, Writing – review & editing. QT: Methodology, Writing – review & editing. LiY: Methodology, Writing – review & editing. XY: Conceptualization, Funding acquisition, Project administration, Writing – original draft. YL: Conceptualization, Funding acquisition, Writing – review & editing.

## Footnotes

^1^https://www.ncbi.nlm.nih.gov/geo/

^2^https://reactome.org

^3^https://genemania.org/

^4^http://biogps.org

^5^https://www.genecards.org
